# How do Iranian older adults define place attachment? a qualitative study

**DOI:** 10.34172/hpp.2021.23

**Published:** 2021-05-19

**Authors:** Zahra Aliakbarzadeh Arani, Nasibeh Zanjari, Ahmad Delbari, Mahshid Foroughan, Gholamreza Ghaedamini Harouni

**Affiliations:** ^1^Aging Research Center, University of Social Welfare and Rehabilitation Sciences, Tehran, Iran; ^2^Spiritual Health Research Center, Qom University of Medical Sciences, Qom, Iran; ^3^Social Welfare Management Research Center, University of Social Welfare and Rehabilitation Sciences, Tehran, Iran

**Keywords:** Aged, Environment, Perception, Qualitative research, Iran

## Abstract

**Background:** Place attachment is the emotional bond between individuals and environment, which seems to increase wellbeing in old age. The purpose of this study was to explore the concept of place attachment from older adults’ perspective.

**Methods:** In this qualitative study, a total of 14 older adults were purposively included in Aran and Bidgool city, Isfahan, Iran. The data were collected using a semi-structured interview and analyzed applying a directed content analysis approach.

**Results:** As participants reported, place attachment meant intensive love, pride, dependency, and familiarity with the environment. Socio-economic attachment was identified as the most prevalent dimension of place attachment, followed by affective, physical, autobiographical, and religious-cultural attachment.

**Conclusion:** Our findings provided a new understanding of place attachment in the context of Iran. The concept of place attachment was identified with a multidimensional nature from Iranian older adults’ perspective. Such a multidimensionality of place attachment should be considered while planning for age-friendly cities or the operationalization of the subject of aging in place, particularly in the developing societies, like Iran.

## Introduction

The world’s population is getting older, and as people age, they build strong affective, cognitive, behavioral, and social bonds to their living environment, which results in intensive forms of place attachment (PA).^1^ PA is an interesting phenomenon investigated in many disciplines. However, there is no unique definition for it,^2^ so there is a diversity in the definition of the concept in different the fields of sociology, geography, psychology, and gerontology.^3,4^ In environmental gerontology, PA means the positive and affective bonds between individual and her/his living environment.^5^ PA is a context-dependent concept that plays an important role in understanding theories and models associated to the well-being and place experiences of individuals.^6^ One of these theories is “person-environment fit”. This theory indicates the degree of fit or match between individuals and their environment and states that the coordination between an environment and a person affects his behavior more than the characteristics of the person or the environment separately. Therefore, to create a sense of security in their life, humans need to belong to an environment and fit with it.^7^

The “theory of attachment” is also an emotional and ethical theory that relates to the attachment of an individual to his surrounding social environment, which results in a security that is effective in the well-being of the individual.^8^

The PA theory is the intersection of two theories; “person-environment fit” and “theory of attachment”. According to the PA theory, the longer the time a person spends in a space, the deeper the meaning he finds in it.^9^ People usually spend more time at home and in the neighborhood after retirement^10^ and increase communication with the community to receive social support.^11^ PA is, therefore, important in older adults,^4^ because it is intensively related to health, function, and well-being in aging, and is a key motivator to improve aging in place.^6^ According to this theory, older adults like to remain in their own homes to maintain their independence and control over their lives, and to preserve their identity and well-being.^12^ Numerous PA models have been proposed, including the functional and emotional two-dimensional model of Stokes and Schumacher (1981) and Proshansky (1978),^13^ the theoretical three-polar framework of “Self, Others, and Environment”,^14^ the three-pole model of “Individual, Social and Natural environment”^15^ and the three-dimensional framework of “People, Places, and Process”.^16^ All these models have been criticized by many researchers.^13^

PA models in older adults have only been studied by Rowles and Berholt. Rowles proposed a three-dimensional theory of insideness in rural older adults.^17^ Berholt also presented a four-dimensional conceptual model of home attachment in a rural area of North Wales which included physical, social, temporal, and psychological components.^18^ These PA models for older adults include important aspects of PA such as the aesthetic aspects, memories, and experiences of living in one place. Although they seem to fit the living situation of older adults in particular time and places and show attachment to a particular rural geographic area such as a village or a remote area, attachment is not discussed in the places such as urban neighborhoods. Therefore, the features and the dimensions of such PA models may not come true for older adults living in urban areas.

Although several previous studies have been done to investigate PA in older adults,^4,11,19-25^ the number of qualitative studies is low and the models and the theories regarding PA in older adults have not well been extracted from the heart of these studies. For example, Rosse in the concept of PA, considers cognitive aspects in addition to certain emotional aspects.^24^ The qualitative findings of the study conducted by Buffel et al in 2014, showed that PA includes two factors “the physical–spatial environment” and “population turnover.”^11^ In the study of Cook et al. in 2007, PA was often expressed in terms of the sense of place, memories, social connections, and familiarity with the environment.^19^ In the study of Wanka in 2017, PA was assessed from the perspective of engagement with the neighborhood.^25^ Based on the findings of these studies, it is clear that even in gerontology, there are no uniform perception of PA. This lack requires further research, especially qualitative studies, to extract the concepts and the meanings from the heart of older adults’ conversations and experiences. Besides generating evidence-based models and theories, the results of such studies can also be used in health planning for older adults and aging in place discussions. Thus, our aim in this article was to explore the concept of PA from the viewpoints of Iranian older adults.

## Materials and Methods

### Participants

In this article, we presented the findings of a qualitative study as the first phase of a mixed-method study on PA among Iranian older adults. Sampling was performed from “community-dwelling older adults” in Aran and Bidgool city, one of the ancient cities of Isfahan province, Iran. In this city, a majority of older adults live in the old parts of the city, the areas where is trying to be rebuilt by the municipality.

The method of selecting participants was purposeful sampling. To achieve maximum sampling variation, we tried to sample male and female older adults from different socioeconomic status and age groups, living in different parts of the city, with diverse length of residence. Participants were interviewed at their own homes or in places they suggested, such as a park or mosque. Data were collected after obtaining informed consent form from all participants. Data saturation occurred after analyzing 14 interviews. The inclusion criteria were consent to participate in the study, with 60 years of age and older, earning a score higher than 7 in the Persian version of the Abbreviated Mental Test (AMT),^26^ willingness to participate in the study, being a resident in Aran and Bidgool city for more than 5 years and ability to communicate.

Nine males and five females were interviewed. The participants were at the range of 60 to 88 years of age with a mean of 70 (SD = 5.07). About 78% of the participants were married, 42% were illiterate and 35% were still in the labor market (Table 1).

### Procedure

To analyze data, the “directed content analysis” method was used. The team of research extracted the main dimensions of PA concept (physical, social, economic, affective, and autobiographical attachment) according to the result of a primary literature review in older adults, and followed them in interviews and data analysis, as a base. Older adults were interviewed through a semi-structured interview in the Persian language by the first author of this article (PhD student, female) and after 14 interviews, theoretical saturation was obtained. The average time of each face-to-face interview was 1 hour and 10 minutes; interviews were recorded and were then transcribed verbatim. An interview guide including the following questions was applied: “Which neighborhood do you belong to?”, “What memories do you have from the neighborhood where you live?”, “Which places in life have you been attached to and why?”, “Which neighborhoods did you most love and why?”, “Where do you like to spend the rest of your life?”, “What is the meaning of the term “PA” from your point of view?”. Participants were also asked to express their views on the main dimensions of PA (including physical, social, economic, affective, and autobiographical) obtained from literature review. Besides, some probing questions were used to specifically explore the experiences and perceptions of participants about PA.

### Data analysis

To make a conceptual framework, a scoping review of previous studies was done, based on which, PA contains five main categories of physical, social, economic, affective, and autobiographical attachment. Main categories included 16 subcategories. Key concepts of this framework were used to design interview guide and data analysis.

Then, MAXQDA10 software (https://maxqda-10.software.informer.com) was used to manage the textual data during data analysis. The coding process was done by the first author. Each written word and phrase was considered as a unit of analysis. Based on the designed framework, five main dimensions were obtained after combining the primary codes into subcategories and the subcategories into the main categories. New main categories and/or subcategories from Iranian context were added to the conceptual framework derived from the literature review phase.

The scoping review helped to establish trustworthiness of the study. The researchers tried to increase their validity with prolonged involvement and complete immersion in the data. In addition, member checks were done by some of the participants to confirm that the results were according to their experiences and perceptions. Findings were also rechecked by experts. Characteristics of participants and their quotations were descripted for more reliability.

## Results

Eighteen subcategories and five main categories including “socio-economic attachment”, “affective attachment”, “physical attachment”, “autobiographical attachment,” and “cultural-religious attachment” emerged from analysis of data. Socio-economic attachment was the most prevalent dimension of PA (33.6% of the codes). The second dimension of PA was affective attachment. This category was seen in 24.1% of the codes.

As Table 2 shows, the third dimension of PA was the physical attachment that included 19% of the codes.

The fourth dimension of PA, autobiographical attachment, was seen in 15.8% of the codes. Finally, cultural-religious attachment as the fifth dimension of PA was mentioned in 7.3% of the codes.

Also, as is shown in Table 2, among 18 subcategories, emotional bonding/dependence was the most common (21.2%) indicator of PA, followed by family bonding (11.7%), community integration (11%), and aesthetics (9.9%). In the following, the main categories of PA are discussed.

### Socio-economic attachment

The socio-economic attachment was the most common dimension of PA from older adults’ viewpoint. Socio-economic attachment comprised five subcategories, namely, “Family bonding”, “Community integration”, “Demographic characteristics of the neighborhood”, “Feeling of Security” and “Affordability”.

*Family bonding*

Family bonding was the most important subcategory of socio-economic attachment. One of the participants said:

*“The reason for belonging to my mother’s neighborhood is just my mother herself” (P11).*

*Community integration*

In the subcategory of “community integration”, participants defined PA as belonging to a social network and having social support. The social network was also defined by the older adults as having a relationship with family, relatives and friends, interpersonal familiarity, friendly bonding, and communication with the neighbors. The presence of neighbors in a person’s social network is very important because sometimes a good neighbor in the neighborhood for older adults is closer and more supportive than many family members and relatives. Also, older adults expressed PA as being active, doing voluntary activities and engagement with society. Social participation and involvement in the local community indicated that an individual perceives himself/herself as a part of the neighborhood and the situation and fate of the neighborhood are important for him/her.

*Demographic characteristics of the neighborhood*

As participants perceived, the “demographic characteristics of the neighborhood” meant the demographic composition of the neighborhood, such as ethnicity and neighborhood development status, and participants pointed out to loving the neighborhood’s population composition, belonging to a neighborhood without any stranger and living in a developed neighborhood.

*Feeling of security*

Participants defined the “feeling of security” as being secure and belonging to the neighborhood without urban problems such as robbery. Having privacy was one of the initial codes of social security which was noted by a majority of the older adults. One of them expressed:

*“I love my neighborhood because my privacy is kept here. No one interferes with anyone’s work and the neighbors don’t disturb each other” (P4)*.

*Affordability*

The last subcategory of socio-economic attachment was “affordability”. From older people’s perspective, this concept was related to economic living in a place, because prices should be affordable for their needed items in the neighborhood. Some of participants reported that they did not have enough money to change their home. Most of the time, this attachment shows a kind of compulsion for older adults because they have to be dependent on their place, due to poor economic status, and as some participants stated they would change their living place if they had enough money.

### Affective attachment

Affective attachment was the second main dimension of PA. This category included two subcategories: “Emotional bonding/dependency” and “Familiarity with the environment”.

*Emotional bonding/dependency*

In the subcategory of “emotional bonding/dependency”, taking into account home as a valuable place, usability of it in the future, and having compassion for home repair were indicative of PA among participants. For example, a 62-year-old man mentioned:

*“One of my hopes is for my home to be preserved after my death. Because I was a teacher, I would personally like my home to be used as a school after my death” (P6).*

To be comfortable in one’s own home and neighborhood was stated by many participants. Because these places are where they live and they get used to it, so they feel comfortable there. In one case, an 83-year-old woman stated:

*“After all, one’s own home is something else. He/She get used to his/her home and is more comfortable. Suppose I am at my daughter’s house, because I have urinary incontinence, when my daughter wants to change my position, she says, “Wow you have made everywhere dirty.” so I get very upset. But if I were in my own home, I would say, that’s okay. I can’t control it” (P1).*

Some other initial codes obtained from our study were pride in the neighborhood, interest in paternal land, the ground of home as a homeland, and missing home and neighborhood. These codes were not in the conceptual framework. One participant stated:

*“Whenever someone asks me, which neighborhood do you belong in? I say I am the child of Ziredeh” (name of the neighborhood) and I am proud of it “(P6).*

One of the older adults stated:

*“The attachment that I feel here, I have nowhere else. If they give me a lot of money and say change your home, I wouldn’t accept. If they give me a house that looks like a palace, I wouldn’t move “(P14).*

Some codes were about the links between a person and his/her property and affairs at home. As one of the older adults stated:

*“Everything in this home is mine. The wall, the garden, the faucet, and the fish pool are mine and under my control. When the faucet is leaking, I’m aware of it. If this home has something good, it’s good for me and if it breaks down, its trouble is mine “(P3).*

*Familiarity with the environment*

In the subcategory of “familiarity with the environment” codes such as familiarity to a home and neighborhood, independence and autonomy, getting used to the physic of an environment, and having a plan in daily life at home were mentioned. One of participants said:

*“This is my place. I feel calm. Elsewhere, I’m confused. I get confused when I go to my children’s homes “(P2).*

### Physical attachment

Physical attachment was the third most frequent dimension of PA. “Aesthetics”, “Accessibility”, “Old age-appropriateness” and “Bonding to nature” were the subcategories.

*Aesthetics*

In this study, “aesthetics” was the most important component of physical attachment. Lighting and outdoor beauty of home and neighborhoods, having enough space at home, and usability of all home spaces were the expressed codes by older adults. A 70 -year-old participant noted:

*“Our home is small but it is enough for us. I love it because it meets our needs. We are using the most of its space”(P10).*

*Accessibility*

“Accessibility” was the second subcategory of physical attachment. Easy access to favorite places, having facilities at home, having welfare, close to downtown, and proximity to the place of worship and mosque were the mostly reported notes among participants.

*“I won’t move anymore and I won’t choose any other place. I want to be here, downtown. Everything is close, bakery, butcher, fruit shop. One brick of this home is more valuable than every home in an area with poor facilities “(P10).*

*Old age-appropriateness*

“Old age appropriateness” was another subcategory emerged from the analysis of data. Safety, expecting aging in place in the future, opportunities for the presence of older adults in the community, age-appropriate services in the neighborhood, and respect for older adults were some of the cases reported by participants. Attitude of an older adult towards safety and old age-appropriateness was expressed in this way:

*“Our neighborhood doesn’t have a lot of alleyways, and its alleyways and streets are flat, and when people see older people, they respect and help them “(P13).*

*“A retirement home is set up in our neighborhood. Some experts are present there to lecture about health, nutrition, and depression. Doctor’s visit is free too, I try to go there and use it”(P11).*

*Bonding to nature*

“Bonding to nature” as another subcategory of physical attachment, included the perceived importance of an environment in terms of health and well-being, physical space, and the beautiful view of the home and the good atmosphere of the neighborhood.

### Autobiographical attachment

The autobiographical attachment category comprised two subcategories, namely, “Temporal and historical bonding” and “Individual memories”. Autobiographic attachment is an important factor in PA because it relates the place to personal identity.

*Temporal and historical bonding*

“Temporal and historical bonding” was about the history of living at home and the neighborhood, birthplace, long periods of living/working in one place and talking about everything related to the past and present of the place, its changes and the happenings over a long period of time.

*Individual memories*

“Individual memories” was another subcategory in the dimension of autobiographical attachment. This subcategory indicated all memories, symbols and reminders of memories in the home and neighborhood environment. This subcategory was one of the most beautiful parts of PA that was pointed out by many seniors. As one example:

*“All my childhood memories are based on the “Washad” neighborhood and can be recalled in an instant. Washad is my birthplace and hometown. When the name of a sister or a brother comes up, the memories of my father’s home are revived. The memories of friends or neighbors come to mind when we hear their name or look at the neighborhood. It is true that many places and streets in Washad have changed, but its symbols are still there “(P11).*

### Cultural – religious attachment

“Cultural - religious attachment” was the last dimension of PA and a new concept in Iranian older adults.

*Rite bonding*

“Rite bonding” such as proximity to a mosque, holy place, and place of worship, attachment to religious ceremonies, and spirituality of the neighborhood was another important aspect of PA, especially in the religious context of Iran. An 82-year-old man explained:

*“Even if I have to move from my home and leave this neighborhood, I will return to this neighborhood to participate in religious ceremonies “(P12).*

*Cultural bonding*

“Cultural bonding” was another subcategory that older adults mentioned. The highly accepted culture of neighborhood people, homogeneity of families’ culture, and the cultural originality of the neighborhood were mentioned by participants in related to PA, as quoted below:

*“I love my neighborhood because it has originality. The people of this neighborhood are more culturally advanced rather than all other neighborhoods “(P13).*

## Discussion

In this qualitative study we explored the Iranian older adults’ viewpoints about PA. Our participants perceived PA as a phenomenon with multidimensional nature. Socio-economic attachment was the most prevalent dimension of PA, followed by affective attachment, physical attachment, autobiographical attachment, and cultural-religious attachment. Therefore, PA was identified as a multidimensional concept that shows the bonding between people and their particular places,^2^ as reported in several previous studies.^15,16,20,27^

Generally, in the current study, PA in older adults means intensive love, pride, dependency, and familiarity with the environment. Among the subcategories of PA, “emotional bonding/dependence” was identified as the most frequently mentioned issue by older adults, and included a variety of codes, like “considering value for home” and “compassion for home repair” as most commonly reports. Older people often reside in dilapidated and ancient homes which may require extensive repairs or rebuilding.^28^ Loving their living place makes the older adults worry about these deteriorations and, as most seniors pointed out, they prefer to repair their home, because they value their place of residence and are unwilling to replace it. Due to the common problems that usually occur in old age, such as frequent urination or musculoskeletal problems, the older people like to spend their old age in their own homes with comfort and independence, because they are familiar with the environment and have gotten used to it.

Among all dimensions of PA, the most prevalent domain was social dimension, which includes community and social support issues. Family bonding was also an important subcategory of PA in the “socio-economic attachment” dimension. From Raymond’s perspective, “family bonding refers to connections to places reliant on family relationships that may relate to family history, interests, concerns, belongingness, and membership.”^15^ Family bonding makes it possible to nurture relationships with family, and in the present community, it was defined in the form of having a family social network, intergenerational support of older adults and children, and having individuals of the same generation living in one’s neighborhood. Among the domains of PA, family bonding was an issue with particular dominance, to the extent that the older adults were willing to relocate from their current place of residence just to be close to their children. This finding is consistent with those reported by Lies et al, who reported family bonding as the most dominant dimension of PA in older adults, after friend and nature bonding.^20^ Compared to developed communities, in the Iranian society, the importance of family support especially in small towns is greater, due to their particular culture.^29^ In the Iranian culture, family discussion and aging in the family has a particular value and, as studies show, supporting older adults by families and their children is an important factor of successful aging.^30^ Thus, the socio-cultural domain of PA has an important role in conceptualizing the phenomenon.

Among older adults participated in the present study, physical dimension of place was a significant issue. In old age, environmental accessibility, age-friendliness and old age-appropriateness of place is necessary. Older adults choose places or change their environment to suit their circumstances. According to the theory of person-environment fit, people have an inherent need to fit their environment, and look for the environments that are match with their characteristics. Because, they like cohesion, and should control their life and reduce uncertainty. They also need attachment, happiness and life satisfaction.^7^

Autobiographical attachment was another main category that usually results in strong attachments to place among older adults. In previous studies, this kind of attachment was conceptualized in different words, like temporal attachment,^31^ place memory^32^ and autobiographical insideness.^17^ This dimension included domains of “temporal-historical bonding” and “individual memories”, and is associated to age, which means that getting older leads to higher level of attachment to place memory. Older adults, in our study, defined this concept as belonging to birthplace, long periods of living or working in one place, all memories or reminders of memories in home and neighborhood, and included historical and temporal issues. Considering that the older adults lived in the same home for a long time, cognitive and emotional aspects of the meaning of place are mostly associated to biography.^33^

Cultural – religious attachment that emerged in the subcategories of rite bonding and cultural bonding in the present study, is an exactly context-dependent component. Religious beliefs and rituals and spirituality in a religious country like Iran, play important role in the structure of PA especially among older adults. So, they are firmly attached to the places that the religious and holy elements, such as mosques, exist in their neighborhood.

The term PA, despite its context and culture-dependent nature, was not so evident in the Iranian seniors’ public conversations, but was common in the life of a majority of our participants. One of participants stated that “*the term PA means we love our place and want to live there. We fell in love with it. Wherever we go, after one or two hours, we want to go back home*”, and another participant stated that “*every bird has a nest; where it calms down, my place (my home and neighborhood) is my nest, so I’m attached to it*”.

## Conclusion

This study explored the perceptions of Iranian older adults of PA. The concept of home and neighborhood attachment meant intense love, pride, dependence and familiarity to the environment and relied on different components of place such as family connection, aesthetic, safety, security, environmental characteristics and memories. This study identified four dimensions of PA (socio-economic, affective, physical, and autobiographical) that covered and confirmed the conceptual framework of the previous findings. The new category of cultural-religious attachment was also obtained as the new and contextual dimension. The findings of this study provided a new understanding of PA in Iranian context which emphasize family bonding in such communities. This study highlights some new meanings of PA, such as pride in the neighborhood and interest in paternal land, as well as the ground of home as a homeland and heritage.

Our findings provided a new understanding of PA in the context of Iran. The concept of PA was identified with a multidimensional nature from Iranian older adults’ perspective. Such a multidimensionality of PA should be considered while planning for age-friendly cities or the operationalization of the subject of aging in place, particularly in the developing societies, like Iran.

## Acknowledgments

We would like to thank all the seniors who contributed to this research.

## Funding

The authors received no financial support for the research, authorship, and/or publication of this article.

## Competing interests

The authors declared no potential conflicts of interest for the research, authorship, and/or publication of this article.

## Ethical approval

This study has been approved by the Research Ethics Committee of the University of Social Welfare and Rehabilitation Sciences (IR.USWR.REC.1398.066) and informed consent was got from all participants. Names and identities of the study participants were not used while analyzing data.

## Authors’ contributions

ZAA, NZ and MF were involved in the conception and designing the study. ZA performed data analysis and interpretation and wrote the manuscript. NZ acted as the corresponding author and supervised the development of work. AD, MF and GhQH helped in data interpretation.

**Table 1:** Demographic profile of the participants in study

**Person**	**Age (y)**	**Gender**	**Marriage status**	**Educational status**	**Job**
1	83	Female	Widowed	Illiterate	Housewife
2	75	Female	Widowed	Illiterate	Housewife
3	63	Female	Married	Elementary	Labor market
4	74	Female	Married	Illiterate	Housewife
5	61	Female	Widowed	Illiterate	Housewife
6	62	Male	Married	Diploma	Labor market
7	70	Male	Married	Bachelor	Retired
8	60	Male	Married	Bachelor	Retired
9	66	Male	Married	Elementary	Retired
10	70	Male	Married	Associate	Retired
11	60	Male	Married	Master	Retired
12	85	Male	Married	Illiterate	Disabled
13	67	Male	Married	Elementary	Retired
14	88	Male	Married	Illiterate	Disabled

**Table 2:** Percentage of participants mentioning each category and subcategory associated with place attachment

**Main categories**	**Subcategories**	**Percentage**	**Percentage**
Socio-economic attachment	Family bonding	11.7	33.6
	Community integration	11.0	
	Demographic characteristics of the neighborhood	5.4	
	Feeling of security	4.4	
	Affordability	1.0	
Affective attachment	Emotional bonding/dependency	21.2	24.1
	Familiarity with the environment	2.8	
Physical attachment	Aesthetics	9.9	19.0
	Accessibility	4.3	
	old age-appropriateness	3.2	
	Bonding to nature	1.5	
Autobiographical attachment	Temporal and historical bonding	9.2	15.8
	Individual memories	6.6	
Cultural – religious attachment	Rite bonding	4.3	7.3
	Cultural bonding	3.0	
